# Therapeutic Assessment of Chloroquine-Primaquine Combined Regimen in Adult Cohort of *Plasmodium vivax* Malaria from Primary Care Centres in Southwestern India

**DOI:** 10.1371/journal.pone.0157666

**Published:** 2016-06-17

**Authors:** Kavitha Saravu, Rishikesh Kumar, Herikudru Ashok, Premananda Kundapura, Veena Kamath, Asha Kamath, Chiranjay Mukhopadhyay

**Affiliations:** 1 Department of Medicine, Kasturba Medical College, Manipal University, Madhav Nagar, Manipal, Karnataka, India; 2 District Health Office, Udupi, Karnataka, India; 3 District Malaria Office, Udupi, Karnataka, India; 4 Department of Community Medicine, Kasturba Medical College, Manipal University, Madhav Nagar, Manipal, India; 5 Department of Microbiology, Kasturba Medical College, Manipal University, Madhav Nagar, Manipal, India; Institut national de la santé et de la recherche médicale - Institut Cochin, FRANCE

## Abstract

**Background:**

Several reports of chloroquine treatment failure and resistance in *Plasmodium vivax* malaria from Southeast Asian countries have been published. Present study was undertaken to assess the efficacy of chloroquine-primaquine (CQ-PQ) combined regimen for the treatment of *P*. *vivax* malaria patients who were catered by the selected primary health centres (PHCs) of Udupi taluk, Udupi district, Karnataka, India.

**Method:**

Five PHCs were selected within Udupi taluk based on probability proportional to size. *In-vivo* therapeutic efficacy assessment of CQ (1500 mg over three days) plus PQ (210 mg over 14 days) regimen was carried out in accordance with the World Health Organization’s protocol of 28 days follow-up among microscopically diagnosed monoinfection *P*. *vivax* cohort.

**Results:**

In total, 161 participants were recruited in the study of which, 155 (96.3%) participants completed till day 28 follow-up, fully complied with the treatment regimen and showed adequate clinical and parasitological response. Loss to follow up was noted with 5 (3.1%) participants and non-compliance with treatment regimen occurred with one participant (0.6%). Glucose-6-phosphate dehydrogenase deficiency (G6PDd, <30% of normal mean activity) was noted among 5 (3.1%) participants and one of them did develop PQ induced dark-brown urination which subsided after PQ discontinuation. G6PDd patients were treated with PQ 45 mg/week for eight weeks while PQ was discontinued in one case with G6PD 1.4 U/g Hb due to complaint of reddish-brown coloured urine by 48 hours of PQ initiation. Nested polymerase chain reaction test revealed 45 (28%) cases as mixed (vivax and falciparum) malaria.

**Conclusions:**

The CQ-PQ combined regimen remains outstandingly effective to treat uncomplicated *P*. *vivax* malaria in Udupi taluk and thus it should continue as first line regimen. For all *P*. *vivax* cases, G6PD screening before PQ administration must be mandatory and made available in all PHCs.

## Introduction

India is one of the main contributors to global burden of *Plasmodium vivax* malaria. The national guideline [1] recommends, chloroquine (CQ) dosage as 25 mg/kg body weight over three days along with primaquine (PQ) as 0.25 mg/kg body weight daily over 14 days for the treatment of all uncomplicated *P*. *vivax* malaria in non-pregnant adults and children aged over one year with normal glucose-6-phosphate dehydrogenase (G6PD) activity. While there have been a few accounts [2–4] of CQ failure with *P*. *vivax* from India, only three such cases dating back to the year 1995–96 reported [5, 6] failure despite adequate blood levels of CQ. *In-vivo* clinical evaluation remains the mainstay of monitoring the antimalarial therapeutic efficacy in *P*. *vivax* malaria and should be employed once every two years [7, 8]. Ideally PQ administration should be withheld until 28 days while carrying out therapeutic trial with CQ in *P*. *vivax* malaria. Nonetheless, assessment of CQ-PQ combined regimen is permitted where regional policy mandates administering PQ with CQ [8].

In Karnataka state, India, *P*. *vivax* imparts above 70% to annual malaria burden [9]. Notably, Udupi and Dakshina Kannada districts remain one of the most fiercely active malaria transmission zones in Karnataka state and they impart in majority to the annual malaria burden in the state (source: District Vector Borne Disease Control Office (DVBDCO), Udupi). Malaria transmission traverses across a year in the district and Udupi taluk contributes utmost to the annual malaria burden in the district. Regional malaria transmission is mainly confined to urban and peri-urban sectors but remain sparse across rural sectors. Mostly, migrant labourers of many on-going mega construction projects across urban and peri-urban sectors in Udupi taluk and to some extent, travellers/workers from other malaria endemic places add to the burden of regional malaria (source: DVBDCO, Udupi). There lacks a published evidence on the efficacy of CQ-PQ regimen among *P*. *vivax* malaria patients being catered by the primary health centres (PHCs) of Udupi district. These regional characteristics portray Udupi taluk as a unique sentinel site to study the efficacy of antimalarial regimen. Present study was conducted to determine the therapeutic efficacy of CQ-PQ combined regimen among adult patients catered by the selected PHCs of Udupi taluk, Udupi district, Karnataka, India as per the WHO’s protocol [8].

## Methods

### Study Design and Population

A prospective cohort study was conducted among microscopically proven symptomatic monoinfection *P*. *vivax* patients aged ≥18 years attending five primary health centres (PHCs) selected within Udupi taluk, Udupi District, Karnataka, India from November 2012 till August 2015. Patients were excluded if they had mixed malaria detected by microscopy or prior antimalarial medication before presentation or pregnancy or unwillingness to provide a written informed consent.

### Study Setting

**Fig 1:**
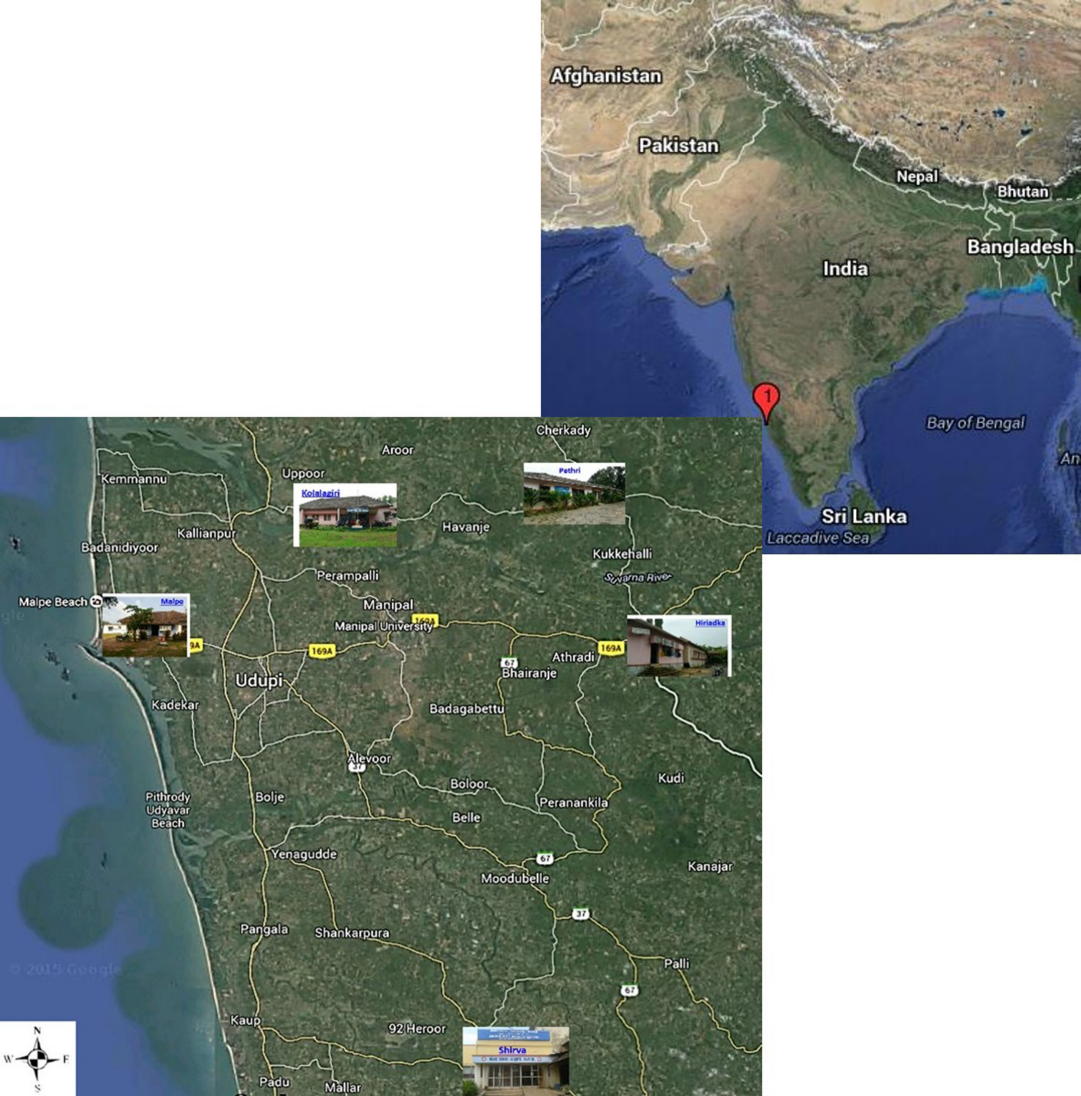
Geographical location of PHCs within Udupi taluk, Karnataka, India. [Image: USGS EROS (Earth Resources Observatory and Science (EROS) Center) (public domain): http://earthexplorer.usgs.gov/].

**Fig 2:**
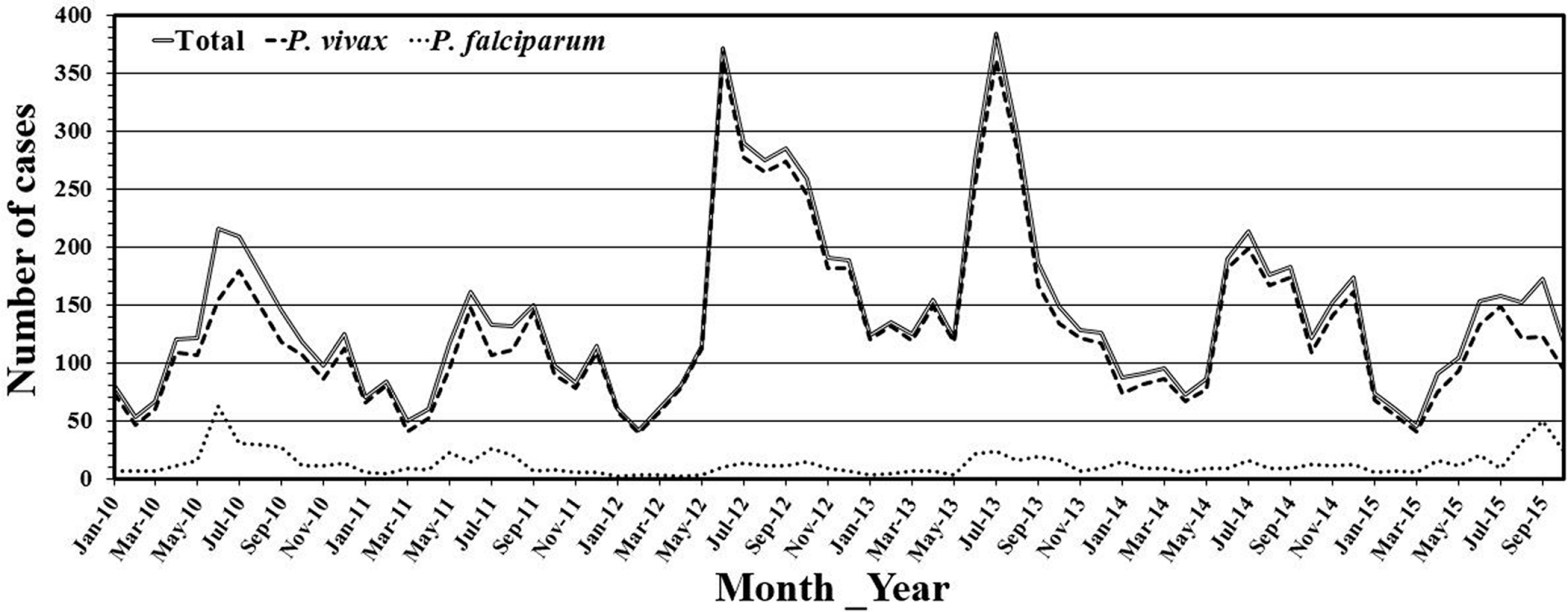
Monthly incidences of malaria cases from January 2010 till October 2015 in Udupi district, Karnataka, India.

Fig 1 depicts the geographical locations of selected PHCs in Udupi taluk. Udupi district is located amid 13°32'24.43" north latitude and 74°52'26.78" east longitude. A tropical monsoon climate prevails and the district records over 4000mm annual rainfall, mostly between June to October. Malaria incidence is recorded throughout a year in Udupi district, peaks during June to July and December, Fig 2. However, a change in trend was observed in the number of total malaria cases reported between July to December from the year 2012 to 2014, this could be attributed to the varying pattern of rain fall. Further exploration into the association of malaria incidence with climatic conditions is required.

Over the past decade, continuing urbanization has led to human migrations and sustenance of malaria transmission in urban and peri-urban sectors of Udupi district. Administratively, the district is delineated into three talukas viz. Udupi, Kundapura and Karkala. Udupi taluk records highest annual malaria cases in the district. The mean annual parasite incidence (API) per 1000 risk population in Udupi taluk over last three years (2012–14) is estimated to be 2.5 as compared to 1.1 of Karkala taluk and 0.3 of Kundapura taluk (source: DVBDCO, Udupi). The foci of local malaria sustenance are in and around rapidly urbanizing sectors. The catchment areas of five selected PHCs mostly include rural areas except, Hiriadka PHCs wherein rapid urbanization and mega-construction projects are going on in different sectors around Manipal for over a decade. The Malpe PHC includes peri-urban areas having shipping and fishery industry.

### Ethics Statement

Study was initiated after obtaining the ethical clearance and permission from concerned organizations. Ethical approval (IEC 193/2011) was obtained from the institutional ethics committee of Kasturba Medical College and Kasturba Hospital, Manipal University, Manipal. Thereafter, an official permission was obtained from the Directorate of Health and Family Welfare Services, Anandarao Circle, Bangalore (letter no. NVBDCP/LAB/30/2012-13). Participation in the study was strictly voluntary and each participant had privilege to retract their participation any time throughout the study. Participants’ identification and study data were kept anonymized.

### Selection of Primary Health Centres and Sample Size Calculation

Of total 22 PHCs across Udupi taluk in the year 2012, five PHCs (Malpe, Kolalagiri, Pethri, Shirva and Hiriadka) were selected based on probability proportional to size. Assuming the proportion of CQ-PQ combined regimen’s failure to be 5%, for a desired precision of 5% and confidence interval of 95%, a cohort of 73 microscopically proven *P*. *vivax* monoinfection cases were required [8]. Furthermore, accommodating a 20% each, expected loss to follow-up and withdrawal/protocol violation, the preferred total was determined as 122.

### Blood Sample Collection and Processing

Prior to administration of antimalarial medications, on day ‘0’, blood samples (one mL) were collected in K_2_ EDTA and lithium heparin vacutainers from each participant. The K_2_ EDTA anticoagulated blood was used for making peripheral smears and estimation of total leukocyte count by Beckman Coulter® LH 750 Haematology Analyzer. Further, DNA extraction was carried out from 200 μL of K_2_ EDTA anticoagulated blood using QIAamp DNA Blood Mini Kit as per the manufacturer’s instructions and stored at -20°C until the conduct of nested polymerase chain reaction (nPCR) test for confirmation of *P*. *vivax* monoinfection. A lithium heparinised blood was used for estimation of G6PD activity by manual spectrophotometric kinetic ‘gold standard’ method [10]. Subsequently on days 2, 3, 7, 14 and 28 both thin and thick smears were obtained by finger capillary puncture. Smears were stained using Leishman’s stain, then examined individually by three microscopists as described elsewhere [11] to ascertain *P*. *vivax* monoinfection and to determine the parasite index (PI) using participants’ total leukocyte count per μL blood.

### Variables

#### Independent variables

Axillary temperature was recorded initially on ‘day 0’ followed by on days 2, 3, 7, 14, 21 and 28. PI was determined as the absolute number of asexual and/or sexual parasites present in one μL of peripheral blood as described previously [11]. Participants’ pertinent details on demography, symptoms and laboratory evaluations were recorded using a customized proforma.

#### Dependent variable

Treatment failure (TF) was deemed as the primary outcome. Therapeutic responses were classified as per WHO’s recommendation for *P*. *vivax* malaria [8].

### Confirmation of *P*. *vivax* Mono-Infection by nPCR Test

Small sub-unit ribosomal RNA was amplified using genus and species-specific primers individually for *P*. *vivax* and *P*. *falciparum*, as described by Snounou *et al*. [12], with few modifications [3].

### Participants’ Treatments

On presentation, all febrile patients were given paracetamol tablet (500 mg) manufactured by Karnataka Antibiotics & Pharmaceuticals Limited (A Govt. of India Enterprise), Plot no. 14, II Phase, Peenya, Bangalore—560058. After microscopic confirmation of *P*. *vivax* malaria, specific antimalarials as supplied and procured by the National Vector Borne Disease Control Programme (NVBDCP), India were dispensed among participants by the staffs of respective PHCs as per the national guideline [1]. Notably, G6PD estimation before PQ administration is not carried out routinely at PHCs across the nation, as it is not mandated by the national guideline [1]. Both CQ and PQ were initiated together on day ‘0’, before the estimation of G6PD activity. Later on, activity of erythrocytes’ G6PD enzyme of every participant was quantified by manual spectrophotometric kinetic ‘gold standard’ method [10]. The reference value of G6PD in India ranges from eight to 18 U/g Hb. Results of all decreased G6PD activity were communicated to the concerned medical officer by 48 hours of initiation of CQ-PQ and then its dose was calibrated accordingly. G6PD deficient (G6PDd, <30% of normal mean activity) patients’ were further treated with 45 mg stat dose of PQ follow by weekly once for subsequent seven weeks. PQ was discontinued in one case with G6PD 1.4 U/g Hb due to complaint of reddish-brown colour urine by 48 hours of PQ initiation. Patients’ compliance to antimalarial medications was determined through group messages over mobile phones and by verifying the emptied drug stripes on every follow-up.

### Statistical Analyses

Continuous variables were summarized as either mean ± standard deviation (SD) or median with interquartile range (IQR). Categorical variables were summarized as frequency with proportion. Log-rank test was performed to compare the survival hazard function between nPCR proven monoinfection *P*. *vivax* and mixed malaria cohorts. A p-value less than 0.05 indicated statistical significance. Data analysis was performed using Statistical Package for the Social Sciences version 15.0 (SPSS, South Asia, Bangalore, India).

## Results

A total of 161 participants satisfying the selection criteria were enrolled including 115 from Hiriadka, 22 from Kolalagiri, 15 from Malpe, seven from Pethri and two from Shirva PHCs. Migrants and construction workers formed majority of the cohort. Pertinent demographic features and laboratory findings of the study cohort have been summarized in Table 1. All participants were aboriginal Indian. No participant was hospitalized during the study.

**Table 1:** Demographic profile of study participants (N = 161) with *P*. *vivax* monoinfection from PHCs of Udupi taluk in southwestern India treated with chloroquine (1500 mg over 3 days) and primaquine^#^ (210 mg over 14 days).

Variables	Mean ± SD / Median (IQR) or Frequency (%)
Age in years	35.9 **±** 13.4
Gender (males)	146 (90.7%)
Past history of malaria	
Never	86 (53.4%)
*P*. *vivax*	65 (40.4%)
*P*. *falciparum*	02 (1.2%)
Species unknown	08 (5.0%)
History of travel to other malaria endemic region in preceding one month	15 (9.3%)
Domiciles	
Migrants	95 (59%)
Native of Udupi district	66 (41%)
Occupations	
Construction worker	92 (57.1%)
Hotel worker	20 (12.4%)
Private security staff	16 (9.9%)
Fishery worker	15 (9.3%)
Others	18 (11.2%)
Duration of fever in days	3 (3, 4)
Axillary temperature at presentation (°F)^a^	99.5 **±** 1.5
Parasite index on day ‘0’ (parasites/μL)*	1184 (970, 1445)
Defervescence duration in days	2.0 **±** 0.08
Treatment failure	None
nPCR proven mixed malaria	45 (28%)

^#**#**^Patients with G6PDd were treated with 45mg/week PQ over eight weeks whereas PQ was stopped in one patient on day three due to G6PD result as 1.4 U/g Hb and complaint of reddish-brown urination.

SD, standard deviation; IQR, interquartile range.

^a^To convert temperature to °C = [°F–32]*5/9.

*Geometric mean with 95% confidence interval of geometric mean.

### Participants’ Treatments and Study Outcomes

Overall, 154 (95.7%) participants completed till day 28 follow-up. CQ phosphate (1500 mg over 3 days, total 10 tablets, each having 150mg CQ base: four each on days ‘zero’ and ‘one’, plus two on day ‘two’) and PQ phosphate (210 mg over 14 days, total 28 tablets, each of 7.5mg PQ: two daily) standard antimalarial regimen for *P*. *vivax* was given to all except five participants whose G6PD activity was deficient (1.4, 3.3, 4.0, 4.0, and 4.6 U/g Hb) i.e. less than 30% of the normal mean (14.8±3.8 U/g Hb) [13]. G6PD data of the study cohort has been summarized in the Table 2 stratified by gender. PQ was withheld after first three doses of 15 mg (by 48 hours of initiation of CQ-PQ), and a weekly dose of 45 mg for next seven weeks was prescribed by concerned medical officers. Notably, the one participant having G6PD activity as 1.4 U/g Hb did complain reddish-brown coloured urine on day ‘three’ and PQ was terminated immediately by medical officer. Mean G6PD activity in the study cohort was determined as 14±4.5 U/g Hb and ranged from 1.4 to 26.0 U/g Hb. A complete compliance for the prescribed CQ-PQ regimen was noted among 95.7% (154/161) participants. Treatment compliance could not be verified with five participants from day ‘seven’ onwards, as they were lost to follow-up (3.1%), however all five had adequate treatment response till day ‘three’.

**Table 2:** Summary of G6PD activity stratified by gender*.

Reference values	Total	Female^♀^	Male^♂^
**Number of cases**	161	15	146
**Mean (U/g Hb)**	14.0	13.1	14.1
**Standard deviation**	4.5	3.4	4.6
**Median (U/g Hb)**	13.8	13.0	13.8
**Range**	1.4–26.0	4.0–18.8	1.4–26.0

*Normal G6PD activity ranges from 8.0 to 18.0 U/g Hb.

^♀^Only one case had G6PD activity less than 8.0 U/g Hb i.e. 4.0 U/g Hb.

^♂^Thirteen cases had G6PD activity less than 8.0 U/g Hb i.e. 1.4, 3.3, 4.0, 4.6, 6.0, 6.3, 6.8, 7.1, 7.6, 7.6, 7.6, 7.6, 7.8 U/g Hb.

Protocol violation/withdrawal was noted with one (0.6%) participants, as he discontinued taking CQ-PQ tablets on day 2. The G6PD activity of the participant was normal (10.4 U/g Hb) and there were no related adverse effects/complaints. He was afebrile throughout the study duration. Further after counselling by the PHC’s staffs, he took the two remaining CQ tablets on day 3, but denied to resume PQ intake, his blood smears till day 7 remained positive as: 719 parasites/μL on day 0 to 151/μL on day ‘two’ to 58/μL on day ‘three’ to 12/μL on day ‘seven’, and then found to be negative on day 14. Then a rebound of parasitaemia i.e. 186/μL was noted on day 21 without any symptom, patient was again counselled and asked to resume the remaining PQ tablets (24 tablets) with him. The participant did consume 14 PQ tablets (105 mg) by the day 28 and was found to be aparasitaemic.

**Fig 3:**
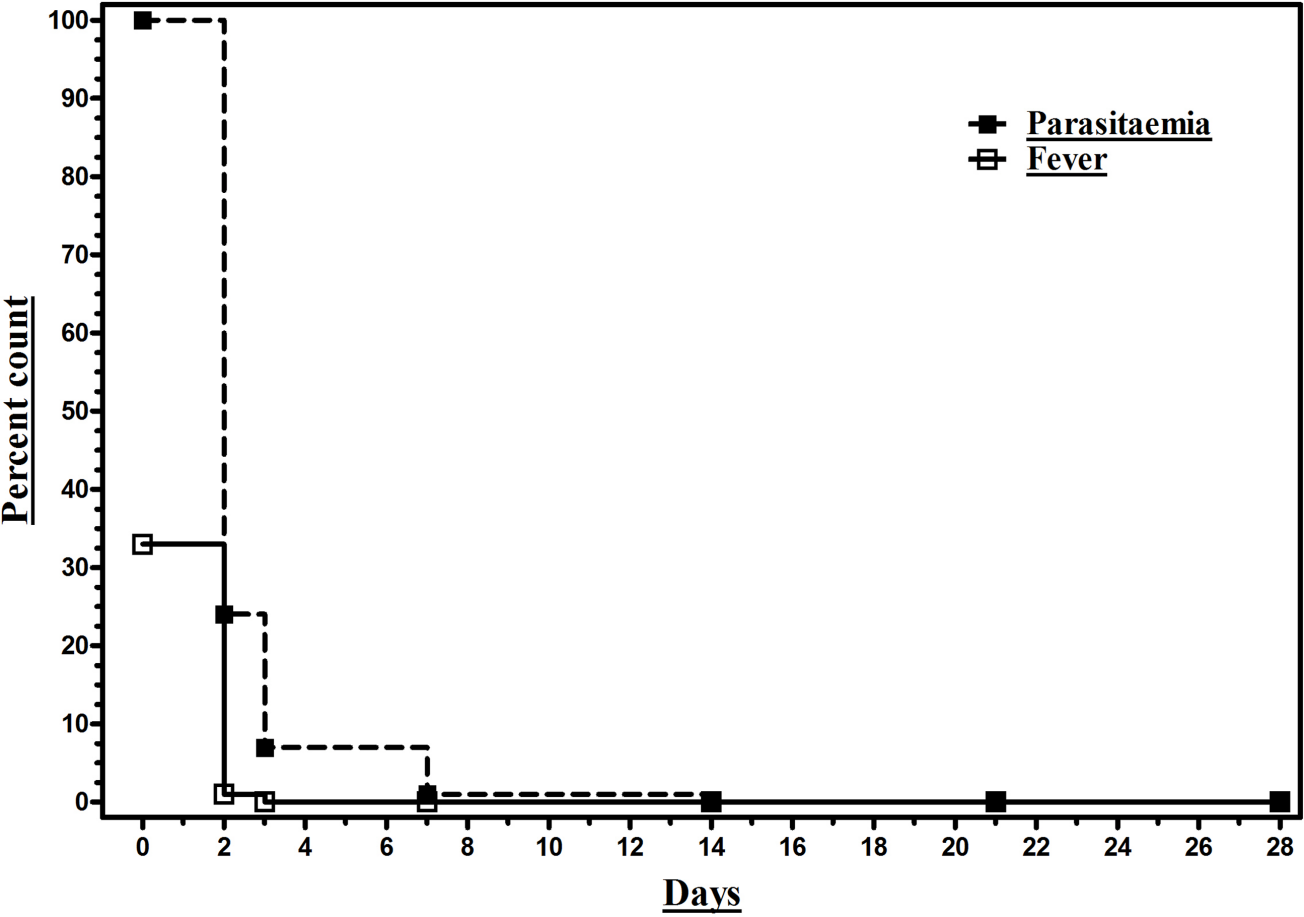
Trend of parasitaemia and fever clearance in a cohort (N = 161) of *P*. *vivax* monoinfection patients from PHCs of Udupi taluk in southwestern India treated with chloroquine (1500 mg over 3 days) and primaquine (210 mg over 14 days).

All participants’ did have fever within preceding three days of presentation, but 67.1% (108/161) were found to be afebrile at presentation and remained so thereafter. Among febrile cohort (32.9%), defervescence duration was noted as two days in 98.1% (52/53) and three days in 1.9% (1/53) participants. Parasites were still observed on day ‘two’ in 24.2% (39/161), on day ‘three’ in 6.8% (11/161), and on day ‘seven’ in 0.6% (1/161) participants (Fig 3). None of the participants met with TF criteria as laid by the WHO [8], excluding the one patient with treatment non-compliance/protocol violation and hence cumulative incidence of therapeutic failure could not be computed. No other adverse outcomes occurred in the study cohort till day 28.

### Nested PCR Results

**Fig 4:**
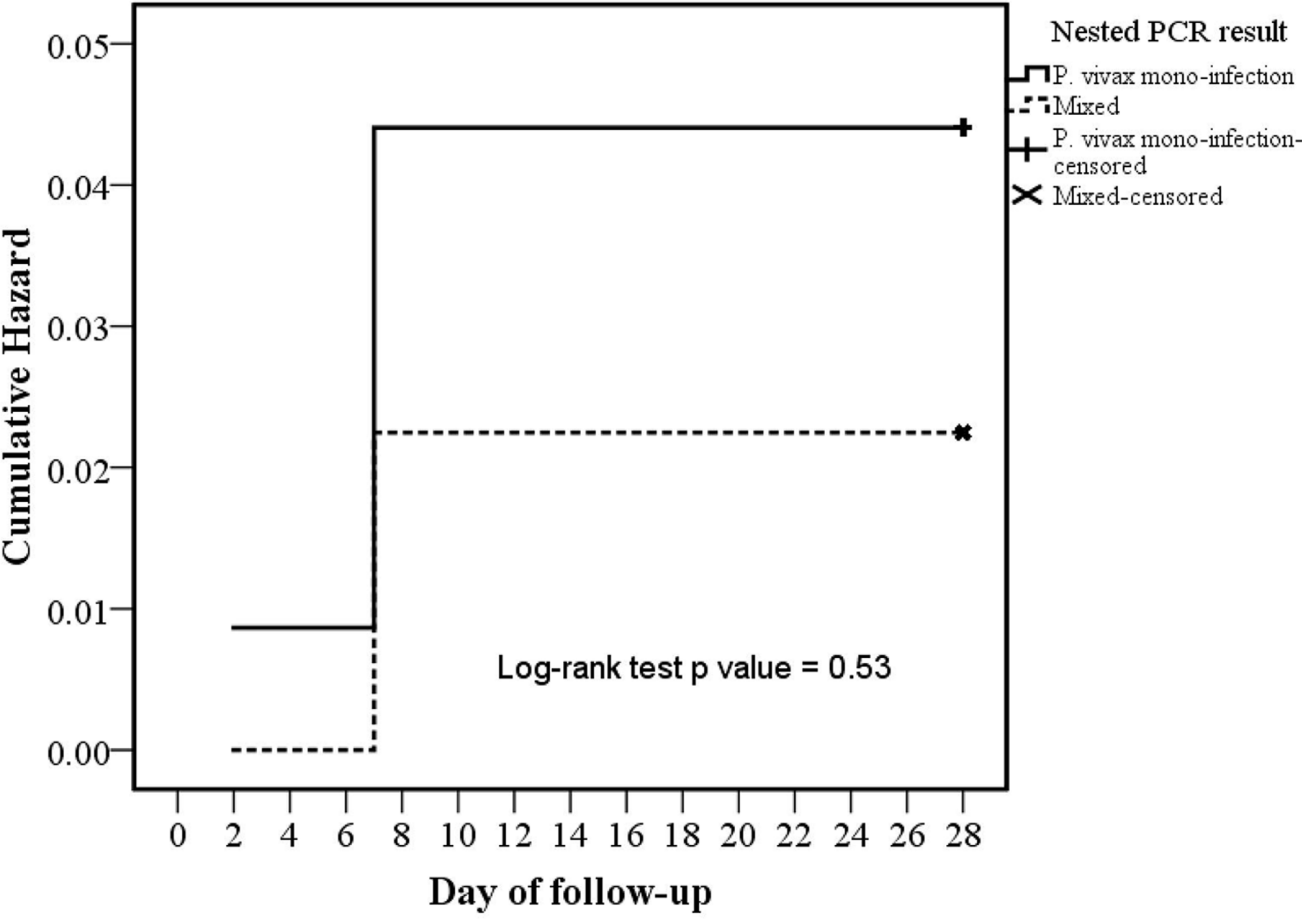
Comparison of survival hazard function between nPCR proven monoinfection *P*. *vivax* and mixed malaria cohorts.

Of total 161 microscopically diagnosed *P*. *vivax* monoinfection, nPCR test confirmed so with 116 (72.1%) cases, however 45 (28%) cases were revealed to be *P*. *vivax* and *P*. *falciparum* mixed infection. The one case with treatment non-compliance/protocol violation did have monoinfection *P*. *vivax* malaria. Fig 4 depicts the comparison of survival hazard function (log-rank test p value = 0.53) between nPCR proven monoinfection *P*. *vivax* and mixed malaria cohorts.

## Discussion

Periodic therapeutic surveillances are the prime requisite to remain vigilant and organized for any untoward emergence of unresponsiveness to existing antimalarial regimens. This study was conducted to assess the efficacy of CQ-PQ combined regimen among symptomatic *P*. *vivax* malaria patients detected by microscopy who were catered by five selected PHCs in Udupi taluk, Udupi District, Karnataka, India.

Male predominance was in accordance with previous series from the study region [3, 11, 14–16]. Majority (90.7%, 146/161) of participants were likely to have contracted malaria infection by local vector inoculation as they did not have any travel history within preceding one month. This finding suggests that, the local malaria is persisting not primarily due to migration, but due to local malaria endemicity. Concrete curing at construction sites provides reservoir for mosquitoes breeding, thereby posing risk for workers and residents of peri-construction sites within mosquito fly range. Also, sum of cases recorded and enrolled into the study from each PHC suggests that, distribution of malaria endemicity in Udupi taluk is patchy and confined to a few regions, mainly in the urban and peri-urban sectors of Hiriadka PHC. This finding may be of interest to regional policy makers to calibrate their planning and implementation of antimalarial campaigns.

Non-compliance with the prescribed antimalarial regimens as evident with one participant, suggests that, in routine practice this might be one of the prime factors for either TF or recrudescence or relapse in *P*. *vivax* malaria. Despite recommendation to supervise PQ therapy over 14 days in national guideline [17], course of PQ treatment is not supervised in routine practice, thus the compliance and success with PQ radical cure remains obscure. Notably, supervised PQ treatment or motivation to comply with PQ dosage has been reported to have better protective efficacy for relapses [18–20]. It is advisable that, in routine practice compliance to prescribed antimalarial drugs must be ascertained in patients for successful malaria control and elimination efforts.

About 100% therapeutic success of CQ-PQ combined regimen as evident from present study is reassuring and in tandem with parallel study result from a tertiary care centre of same region [3] and from Kolkata [21]. Whereas other studies from Mangalore [22], Chennai [23] and Gujarat [24] have also reported ~100% efficacy of CQ monotherapy. Despite this reassuring efficacy of CQ-PQ regimen in the study region, surging CQ resistance in *P*. *vivax* among neighbouring countries viz. Myanmar [17], Thailand [25] and Vietnam [26] poses a threat to India and calls for persistent vigilance.

### Strengths and Limitations

Standard study design with statistically valid cohort size and robust analysis renders the findings of present study reasonably comparable with other series across the globe. Outstanding therapeutic efficacy as evidenced in this study and other parallel study [3] reassures the continuation of CQ-PQ combined regimen for the treatment of uncomplicated *P*. *vivax* malaria in Udupi region. Notwithstanding, G6PD deficiency in both present and parallel study [3] and resulting complication upon PQ administration as noted in present study claims routine mandatory G6PD screening in all *P*. *vivax* cases before PQ administration. Loss to follow-up with five participants and protocol violation with one participant is within anticipated limit i.e. 20% each and was already accounted in sample size, thus these two untoward events do not affect the outcome and conclusion of the study. However, possibility of TF in otherwise adequately responding lost to follow-up cases by day ‘three’ could not be ruled out further day ‘seven’ onwards. Protocol violation due to non-compliance to the treatment regimen precluded the legitimate assessment of therapeutic efficacy in one case. Arguably, one could imply an event of reappearance on day 21 as recrudescence due to CQ blood concentration below 100 ng/mL, the threshold defined for *P*. *vivax* CQ resistance [8]. However, authors did not consider so for the two reasons, first the case was studied in rainy season of July month while regional malaria transmission remains at its peak and reinfection becomes highly probable, besides blood/plasma concentration of CQ was not determined. In this context, it is important to note that, this study underscores the excellent efficacy of CQ-PQ combined regimen but not CQ alone. Also, it is advisable that, future studies should withhold PQ administration till day 28, to assess CQ’s efficacy as per the WHO’s protocol [8].

Revelation of substantially high proportion of mixed malaria cases (28%) by the nPCR test in microscopically diagnosed and otherwise clinically well responded monoinfection *P*. *vivax* cohort imposes question on the implications of nPCR test results. In another parallel study from a tertiary care centre [3], cases of nPCR revealed mixed malaria (6.4%, 8/125) did not have any different clinical consequence. Conversely, one may relate this high proportion of nPCR proven mixed malaria to either residual subpatent parasitaemia due to inadequate treatment of past malaria or subpatent asymptomatic malaria prevailing in the region. In fact, majority (53.4%) of participants of present study never had malaria and 40.4% participants had history of *P*. *vivax* malaria. Thus, inadequate treatment causing residual subpatent parasitaemia as the cause of high mixed malaria diagnosis by nPCR is remote. Hence, it remains plausible that the study region might have high prevalence of asymptomatic subpatent *P*. *falciparum* parasitaemia due to low socio-economic status and baseline immunity, which must be explored further. Whether *P*. *falciparum* isolates in nPCR proven mixed infections were susceptible to CQ-PQ combined regimen, could have been determined only if nPCR test was performed on subsequent follow-up as well. Hence, we cannot rule out the possibility of persisting subpatent asymptomatic *P*. *falciparum* parasitaemia in otherwise ‘adequate clinical and parasitological response’ group. Thus, further exploration to determine susceptibility of CQ-PQ regimen in *P*. *falciparum* isolates from subpatent asymptomatic malaria is justifiable.

## Conclusions

The CQ-PQ combined regimen remains outstandingly effective to treat uncomplicated *P*. *vivax* malaria in Udupi taluk and thus it should continue as first line regimen. For all *P*. *vivax* cases, G6PD screening before PQ administration must be mandatory and available in all PHCs.
